# A Sensitive and Portable Double-Layer Microfluidic Biochip for Harmful Algae Detection

**DOI:** 10.3390/mi13101759

**Published:** 2022-10-18

**Authors:** Ping Li, Le Qiang, Yingkuan Han, Yujin Chu, Jiaoyan Qiu, Fangteng Song, Min Wang, Qihang He, Yunhong Zhang, Mingyuan Sun, Caiwen Li, Shuqun Song, Yun Liu, Lin Han, Yu Zhang

**Affiliations:** 1 Institute of Marine Science and Technology, Shandong University, Qingdao 266000, China; 2 Key Laboratory of Shaanxi Province for Craniofacial Precision Medicine Research, College of Stomatology, Xi’an Jiaotong University, Xi’an 710004, China; 3 Department of Endodontics, Hospital of Stomatology, Xi’an Jiaotong University, Xi’an 710004, China; 4 CAS Key Laboratory of Marine Ecology and Environmental Sciences, Institute of Oceanology, Chinese Academy of Sciences, Qingdao 266071, China

**Keywords:** microfluidic biochip, harmful algal bloom, nuclei acids sensing, environmental warning, photoluminescence detection

## Abstract

Harmful algal blooms (HABs) are common disastrous ecological anomalies in coastal waters. An effective algae monitoring approach is important for natural disaster warning and environmental governance. However, conducting rapid and sensitive detection of multiple algae is still challenging. Here, we designed an ultrasensitive, rapid and portable double-layer microfluidic biochip for the simultaneous quantitative detection of six species of algae. Specific DNA probes based on the 18S ribosomal DNA (18S rDNA) gene fragments of HABs were designed and labeled with the fluorescent molecule cyanine-3 (Cy3). The biochip had multiple graphene oxide (GO) nanosheets-based reaction units, in which GO nanosheets were applied to transfer target DNA to the fluorescence signal through a photoluminescence detection system. The entire detection process of multiple algae was completed within 45 min with the linear range of fluorescence recovery of 0.1 fM–100 nM, and the detection limit reached 108 aM. The proposed approach has a simple detection process and high detection performance and is feasible to conduct accurate detection with matched portable detection equipment. It will have promising applications in marine natural disaster monitoring and environmental care.

## 1. Introduction

Harmful algal blooms (HABs) are caused by the excessive proliferation or aggregation of red tide algae, protozoa or bacteria in the seaweed family under specific conditions [1]. A large number of harmful algal cells floating on the water surface not only reduces the transparency of the water surface, endangering the survival of aquatic animals and plants, but also produce toxins, which accumulate step by step along the food chain, seriously destroying the normal ecological balance of lakes or oceans, endangering global public health [2] and bringing huge economic losses to society [3]. The global economic impact of marine phycotoxins is estimated to be approximately USD 4 billion a year [4]. For monitoring and early warning, the critical algal cell concentration can be set to 10^6^ cells/L [5], but the detection process is still complicated. Several methods for the detection of HABs have been studied [6]. Traditional methods for quantitative detection of harmful algae are mainly based on microscopic identification and counting [7] and molecular biology techniques, which include polymerase chain reaction (PCR), real-time fluorescent quantitative PCR (RT-PCR) [8] and some hybridization techniques [9,10]. Microscope-based counting work requires professional observers because the size of the algae cells under the microscope is too small to see the difference [11], which is challenging to realize accurately quantitative detection of HABs except with assistance of Flow Cytometer [12]. Considered as rapidly developing detection methods, molecular biology detection techniques are based on the specific gene sequence of algae, such as polymerase chain reaction (PCR) and real-time fluorescent quantitative PCR (RT-PCR) [8], but the processes are complex and time-consuming due to multiple cycles of amplification [13]. In order to maximize the fluorescence effect and minimize the cross-reactivity of the probe, a whole-cell hybridization technology was developed [10]. However, the intensity of the fluorescent signal constantly changes along with the diverse expression of RNA in different growth stages of algae cells, thus affecting the detection efficiency of whole-cell hybridization technology [14]. A sandwich hybridization method was developed to detect *Karenia brevis*, and it was found that the signal of *Karenia brevis* in field samples was weaker than that in laboratory detection [9]. In addition, the tedious experimental process could easily lead to the interference of sample DNA or RNA. The Nuclease Protection Assay integrated with the Sandwich Hybridization (NPA-SH) approach was established for *Heterocapsa triquetra* detection through nuclease protection assay sandwich hybridization, with a detection limit of 15,000 cells per milliliter [15]. It has strict requirements for the degree of algae fragmentation [16], which is difficult to achieve in algae samples because of the hard cell walls, such as diatoms. The fully automatic in situ observation technology of algal bloom breaks through the limitations of time and space. Changes of HABs in nearby waters are being recorded by two land-based passive acoustic listening stations (PALS) deployed in Sarasota Bay, Florida [17]. This technique is promising in algae monitoring, except for its expensive equipment, which limits its wide application.

Microfluidic biochip technology is a novel and booming technology widely used in many fields because of its high throughput and detection efficiency [18,19]. In addition, some bumpy sites, such as the ocean, are not conducive to field detection, while the portable characteristics of microfluidics facilitate simple sampling and processing, improving the accuracy of subsequent experiments to some extent. It can also be combined with electrochemical detection [20], field-effect transistor [21] and optical detection to realize high-performance sensing. Furthermore, nanomaterials provide promising applications in biological molecules detection, such as graphene [22,23], graphene oxide (GO) [24,25,26], MoS_2_ [23,27] etc., which have fluorescence resonance energy transfer (FRET) properties. GO contains a large number of oxygen atoms in the form of epoxy, hydroxyl and carboxyl groups, which have excellent dispersibility in aqueous media, and thus exhibit excellent biocompatibility and high optical quenching ability [22,23], so it is promising to integrate GO nanomaterial with microfluidic chips to realize rapid and ultrasensitive detection using simple sensing system.

Here, we developed an ultrasensitive biosensor platform based on a microfluidic biochip and GO nanomaterials to rapidly detect six common HABs. GO was utilized to firstly quench the fluorophore labeled on single-stranded DNA (ssDNA), and after it was hybridized with complementary target ssDNA to form a double-strand DNA (dsDNA), the fluorescence of the fluorophore group was recovered because of its desorption from GO. The fluorescence recovery efficiency of the microfluidic biochip was calculated according to the fluorescence analysis method and the Stern–Volmer equation, thus resulting in the rapid quantitative detection of the specific genes of multiple HABs simultaneously in parallel reaction chambers. When sampling at sea, immobilizing the sample in the microfluidic biochip can simplify its collection and storage in transit without affecting subsequent detection. The low-cost, high accuracy, high throughput and easy-to-operate properties of the microfluidic biochip provide a promising platform for algae monitoring and environmental care.

## 2. Materials and Methods

### 2.1. Materials

Cation buffer included 2.5 mM magnesium chloride (MgCl_2_), 20 mM Tris-HCl buffer (pH 8.0) and 0.5 mM calcium chloride (CaCl_2_), which were purchased from Solarbio Science and Technology Corporation, Beijing, China. SU-8 photoresist was obtained from Xi’an Bona. Polydimethylsiloxane (PDMS) and curing agent were purchased from Dow Corning (Midland, MI, USA). Sodium hydroxide (NaOH), concentrated sulfuric acid (H_2_SO_4_), 30% hydrogen peroxide (H_2_O_2_) and isopropanol (C_3_H_8_O) were supplied by Sinopharm. The glass slides were obtained from Jiangsu Shitai experimental equipment Co., Ltd.

### 2.2. Algae Culture

We selected six HABs that occur frequently or produce toxins, including Amphidinium carterae Hulburt, Alexandrium catenella, Heterosigma akashiwo, Karenia mikimotoi, Prorocentrum lima and Skeletonema costatum, as shown Tables S1 and S2. We cultured them axenically at 19 to 21 °C in f/2 medium in a light:dark (12:12 h) cycle with a photon flux density of 100 μmol/(m^2^s). The culture flasks were shaken 4 times a day to prevent the algae cells from attaching to the wall or sinking.

### 2.3. DNA Extraction and PCR Amplification

**Figure 1:**
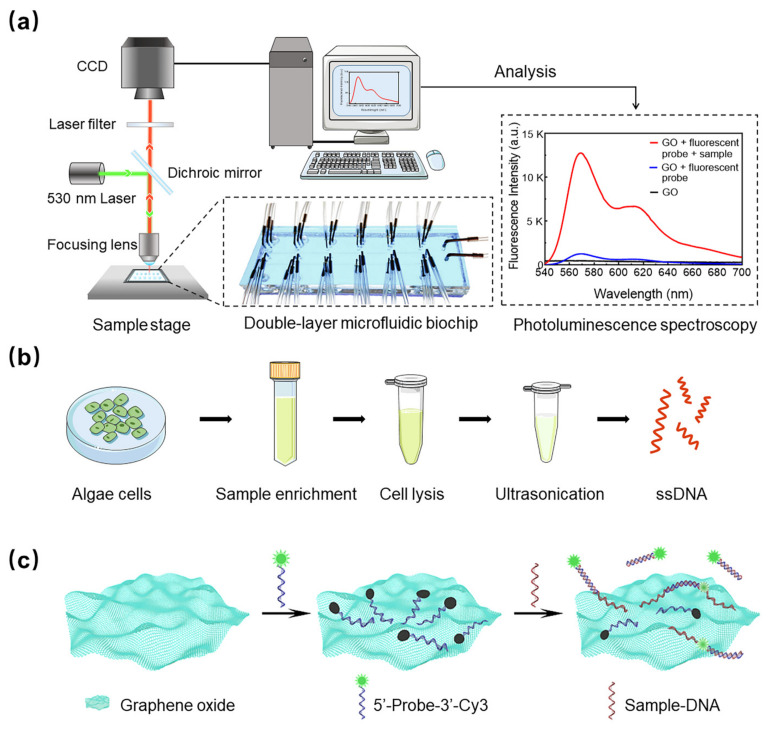
(**a**) Schematic diagram of the double-layer microfluidic biochip sensor. (**b**) Sample treatment process. (**c**) Graphene oxide sensing mechanism.

Refer to our previous experiments [28], the total DNA of algae cells was extracted using the DNeasy^®^ PowerWater^®^ kit produced by QIAGEN (Figure 1b) and was amplified using the universal primer sequence of the 18S rDNA gene of algae (Primer 1:5’-CCGGATCCTGATCCTTCTGCAGGTTCACCTAC-3’, Primer 2:5’-CGAATTCAACCTGGTTGATCCTGCCAGT-3’) [29]. The 18S rDNA gene was chosen because it is highly conserved during evolution and is widely used in eukaryotic algal cells [28]. The PCR reaction was performed on a gradient PCR instrument produced by BIO-RAD. Each 50 µL PCR reaction system contained 25 µL 2 × Super Pfx MasterMix, 2.5 µL 10 µM primer 1, 2.5 µL 10 µM primer 2 and no more than 250 ng template DNA. The PCR amplification protocol was 3 min predegeneration at 98 °C, 35 cycles of denaturation at 98 °C for 10 s, annealing at 52 °C for 1 min, extension at 72 °C for 25 s and then a 7 min extension at 72 °C. After the PCR amplification was completed, 2 µL of the product were placed on a 1.0% low-density agarose gel for electrophoresis (100 V, 40 min). The qualified samples with clear bands were recovered and then sequenced by Sangon Biotech (Shanghai, China) Co., Ltd.

### 2.4. Probe Sequence Design

According to the sequencing results of 18S rDNA, we found a region with large sequence differences between the target algae and other algae in the same genus and different species and used OLIGO 7 primer analysis software to design specific cyanine-3 (Cy3) fluorescent probes for six HABs in this region. The obtained probe sequences were compared again to confirm the specificity of bioinformatics analysis. All oligonucleotide sequences used in this experiment were synthesized by Sangon Biotech (Shanghai, China) Co., Ltd. The designed oligonucleotide sequences are shown in Table S3.

### 2.5. Photoluminescence Detection System

The photoluminescence (PL) chip detection system consists of a 530 nm laser to excite the fluorescence molecule, a common microscope to observe the sample and a spatial filtering acceptor to collect photons from the diffraction limit and suppressed photons outside the detection area, thus removing stray light in the defocused area effectively and obtaining more realistic PL information of the sample. The PL spectrum of the GO-fluorescent probe and GO-fluorescent probe sample was obtained by the Charge Coupled Device (CCD) detector of the PL system, and the peak of the spectrum at 570 nm was taken as the output sensor signal for data analysis.

### 2.6. Chip Design and Fabrication

As shown in Figure S1, the double-layer microfluidic biochip structure was designed with 12 detection units, and they share the same inlet and transporting channel for conjoint regents loading. Each unit has a separate probe and sample loading inlets. The volume of each detection unit is 20 μL. The samples, probe and GO solutions were injected into the chamber through the pression using a syringe. Microfluidic biochips were mainly fabricated based on lithography, and nano-sensing units were integrated into the chips. The fabrication process and actual size of microfluidic biochip production are illustrated in Figures S2–S4. Specifically speaking, SU8-2035 was spin-coated on a clean silicon wafer with a thickness of 30 µm. After prebaking at 65 °C for 3 min and 95 °C for 6 min, the sample was exposed to ultraviolet light for 3s twice. Then, the wafer was baked at 150 °C for 40 min. After development, the designed pattern was displayed. Then, the photoresist pattern on the silicon wafer was further transferred to the chip using the PDMS transfer process. PDMS and curing linker were mixed at a mass ratio of 10:1, and the vacuum-evacuated PDMS was poured into the mold, which was placed in an oven at 80 °C for 1 h to cure and peel off. Holes were punched at the liquid inlet and outlet positions before the upper and lower sides of PDMS were soaked in isopropanol for cleaning and placed in an oven at 80 °C for 1 h. Finally, the two layers of chips were bonded to synthesize a complete double-layer microfluidic biochip, thus completing the design and fabrication of the entire biochip (Figure S4). The 7.5 cm × 2.5 cm glass substrate was used to build up the biochip, and it could be scaled up easily using a larger substrate.

### 2.7. GO Synthesis and Characterization

GO was synthesized by a modified Hummer method [30], and then its material characteristics were performed. X-ray photoelectron spectroscopy (XPS) was carried out on the Thermo Scientific Escalab 250Xi. A FEI Tecnai G2 F20 High-Resolution Transmission Electron Microscope (HRTEM) was used to test the morphology and structure of GO. Fourier Transform Infrared Spectroscopy (FTIR) was carried out on the Thermo Fisher Nicolet iN10. The material composition was expressed by X-ray Diffraction (XRD) and measured by the Bruker D2 PHASER. The UV absorption spectrum was measured by a multi-function microplate detector (BioTek Synergy™ H1, Winooski, VT, USA) with a wavelength range of 200–800 nm. A Renishaw inVia Reflex Raman microscope was used for the measurement of laser Raman spectroscopy using an argon ion beam with a 532 nm wavelength.

### 2.8. Specificity and Sensitivity Tests of Microfluidic Biochip

Briefly, we first used PCR-amplified 18S rDNA fragments for system modeling, during which the purified and pollution-free 18S fragments ensured the accuracy of specificity and sensitivity. Then, we directly detected various HABs in the actual samples by extracting the DNA of the HABs without amplification. Based on the optimization results of our previous experiments [28], 2 µL of 1 µM Cy3-labeled specific probe and 1.2 µL of 0.1 mg/mL GO nanomaterial solution were added into each unit on the biochip through the flow channel at room temperature, followed by incubating for 5 min. Afterwards, 2 µL 18S rDNA target gene of HABs at different concentrations were added, and the system volume was filled up to 20 µL with cation buffer. After incubating at 65 °C on the heating stage for 30 min and slowly cooling to room temperature within 10 min, the microfluidic biochip was scanned by the PL detection system under the excitation wavelength of 530 nm. The final concentration of each Cy3-labeled specific probe was 100 nM, and the GO nanomaterial solution was 6 µg/mL. Each experiment was repeated three times, and a control group of 100 nM Cy3-labeled specific probes was established.

### 2.9. Preparation and Treatment of Mixed Samples

Six species of HABs cells were randomly selected at different concentrations and were continuously diluted 6 times with a gradient of 10, then divided into six parts according to the ratio in Table S4 and mixed well. After that, the total DNA of each sample was extracted and sonicated. To minimize the contamination and complexity of the experiment, PCR amplification and purification experiments were not performed.

### 2.10. Data and Statistical Analysis

OLIGO 7 and DNAman 8 software were used to design and calculate DNA sequences and mass. Three parallel experiments were conducted for each test. Statistical analyses and graphs were generated using Graphpad and Origin software. The calculation method of LOD and the conversion process between algae and DNA concentration were detailed in the supporting documents.

## 3. Results and Discussion

### 3.1. Algae Detection Biochip and System

A double-layer microfluidic biochip and a PL detection system were developed to conduct ultrasensitive detection of harmful algae, as shown in Figure 1a. The variation in representative spectrum peak intensity indicates the concentration of targets in samples and is used to quantify target concentration. The proposed detection approach has a simple sample treatment process, as shown in Figure 1b. The core of the reaction system is based on the quenching effect of GO. Cy3-labeled ssDNA is adsorbed on the GO surface by π-π stacking to quench the fluorescence [31]. The addition of complementary strands paired with ssDNA formed a double strand. The stable hydrogen bond between the duplexes effectively shields the carbon phosphate skeleton and interferes with the interaction between ssDNA and GO, resulting in the duplex’s desorption and fluorescence recovery [32,33]. The fluorescence intensity of the GO or fluorescent probe solution is detected through the PL system. The schematic diagram of the detection process is shown in Figure 1c.

**Figure 2:**
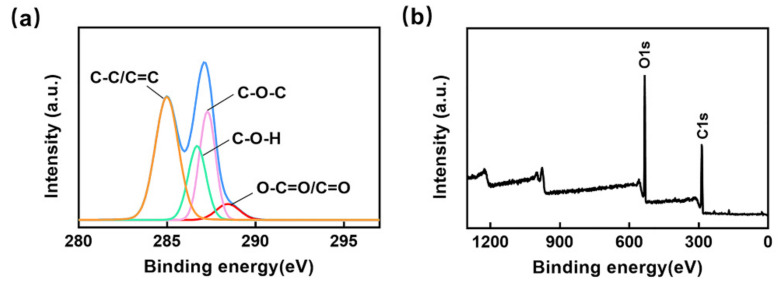
(**a**) XPS image of GO (C1s). (**b**) XPS image of GO (full spectrum).

The material properties of GO affect the fluorescence quenching efficiency, which in turn affects the detection performance of the double-layer microfluidic biochip, so a series of characterizations of GO were conducted. The XPS image of GO is shown in Figure 2a,b. The C1s spectrum of GO could be expressed as the carbon skeleton peak (C-C/C=C) at 284.6 eV, the hydroxyl peak (C-OH) at 286.5 eV, the epoxy group peak (C-O-C) at 287.1 eV and the carboxyl/carbonyl peak at 288.5 eV (O-C-O/C-O). The TEM image in Figure S4a shows the lamellar morphology of GO, and the more enlarged TEM image in Figure S4b shows that the lattice spacing was 0.90 nm, which was consistent with the result of the XRD pattern in Figure S4c. GO had a strong absorption peak of FTIR spectrum at 1053 cm^−1^, which was the C-O stretching vibration peak, 1618 cm^−1^ corresponded to the stretching vibration peak of the C=C framework and 1726 cm^−1^ and 3423 cm^−1^ corresponded to that of C-O and -OH in GO (Figure S4d). The UV-visible spectrum in Figure S4e indicates that the maximum absorption of GO was at 234 nm, which corresponded to the aromatic C=C bonds π-π* transition, and the shoulder surrounding 300 nm corresponded to the aromatic C-O bonds n–π* transition. The Raman spectrum in Figure S5f shows the D peak, G peak and their overtones of GO.

Here, we defined the recovery efficiency of the fluorescent group as the detection performance of a double-layer microfluidic biochip, which is used to create a standard curve for the quantification of algae. The Stern–Volmer equation [34] is usually used to express the relationship between the concentration of the quencher and the fluorescence intensity of the system, and we further deduced and converted this formula in the previous paper [28] to apply to our data analysis. The peak of the PL spectrum at 570 nm was taken into the following formula to determine the recovery efficiency of the fluorophore:

(1) $$\frac{F_{R}}{F_{Q0}}=1+K_{a}\left[Q\right],$$

where *F_R_* is the fluorescence intensity at the representative peak after adding the ssDNA of the sample to be tested; *F_Q_*_0_ is the fluorescence intensity at the representative peak of Cy3-labeled specific fluorescent probe along with GO nanomaterial composite, that is, the fluorescence intensity of the solution after GO nanomaterials quenching the Cy3 fluorophore; *K_a_* is the effective binding constant of the hybridization between the sample, and the specific fluorescent probe; [*Q*] is the concentration of ssDNA of the tested sample.

### 3.2. Detection Performance of Double-Layer Microfluidic Biochip

To investigate the selectivity and sensitivity of a double-layer microfluidic biochip to detect the 18S rDNA genes of the six HABs, here the purified PCR products were used as a standard target sample. First, PCR dsDNA was melted into ssDNA, and its concentration was tested. The excitation wavelength of PL detection was 530 nm, while the emission wavelength peaks at 570 nm of the solution with 18S rDNA genes of different concentrations (from 10^−4^ pM to 10^5^ pM) were regarded as *F_R_*, and without target genes were regarded as *F*_*Q*0_.

**Figure 3:**
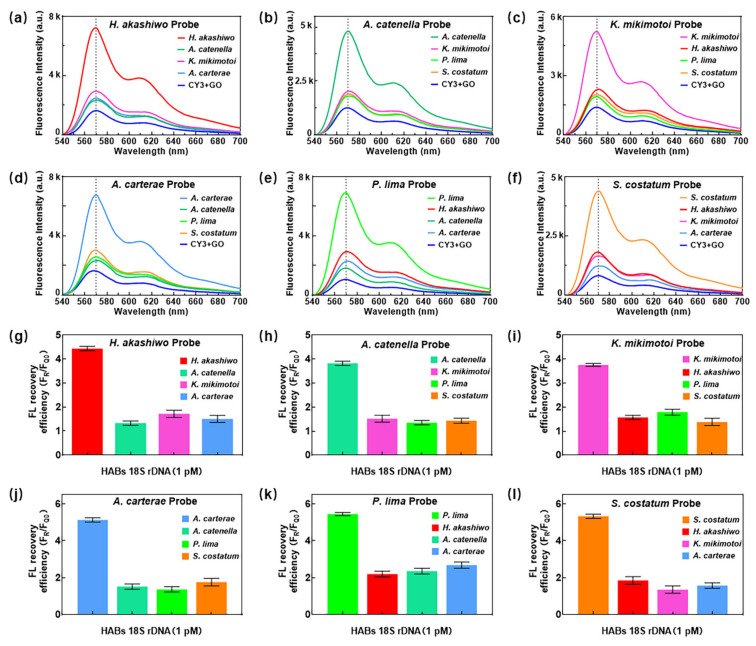
(**a**–**f**) Selectivity test and (**g**–**l**) fluorescence recovery efficiency of six HABs using the double-layer microfluidic biochip and PL system.

Figure 3 reveals the PL spectrum and fluorescence recovery efficiency, expressed as *F_R_*/*F*_*Q*0_, of the specific detection of six HABs. The PL spectrum peak of the 18S rDNA gene of *Heterosigma akashiwa* at 570 nm was significantly higher in the reaction unit with *Heterosigma akashiwa* probes than that of the other algae cells, and it showed similar results to other probes designed for other five algae, as in Figure 3a–f. According to the equation of fluorescence recovery efficiency, it is defined as the ratio of peak intensity at 570 nm after sample loading over that before sample loading. Figure 3g–l presents that the fluorescence recovery efficiency of matched algae to the probes is much higher than that of other algae, a similar trend to Figure 3a–f. At the same time, the fluorescence recovery efficiency of the *Heterosigma akashiwa* 18S rDNA gene was significantly higher than that of the 18S rDNA genes of the other three algae cells (Figure 3g). Both the PL spectrum and fluorescence recovery efficiency indicate excellent selectivity of the proposed detection system: *Heterosigma akashiwa* is selective from 10^−4^ pM to 10^5^ pM, while *Alexandrium catenella* from 10^−2^ pM to 10^5^ pM, and the others are selective from 10^−3^ pM to 10^5^ pM.

**Figure 4:**
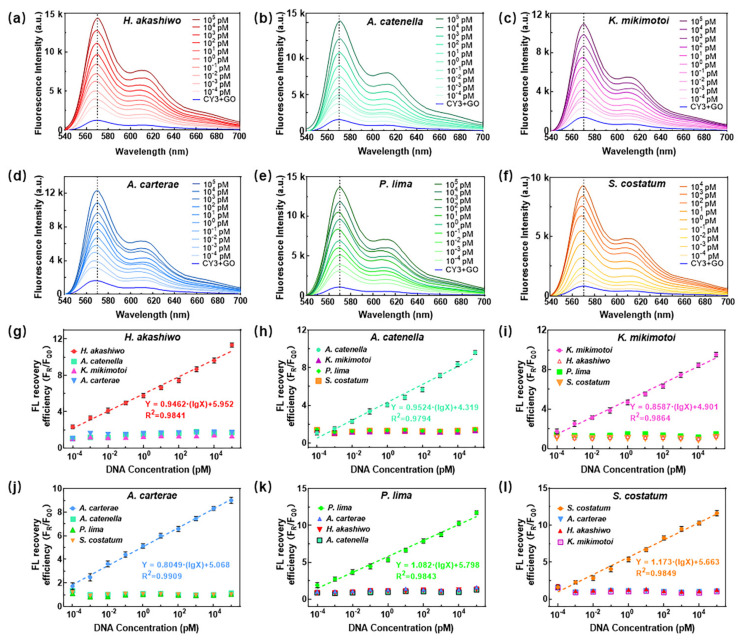
(**a**–**f**) PL spectrum of 18S rDNA gene of six algae with different concentrations. (**g**–**l**) Fluorescence recovery efficiency dependence on concentration of 18S rDNA gene of six HABs.

To obtain the standard fluorescence curves of the six HABs for quantitative detection, the PL spectra and fluorescence recovery efficiency of the target genes with different concentrations were measured through the biochip and PL system, as shown in Figure 4. The excitation wavelength was 530 nm, while the emission spectrum had two peaks near 570 nm and 610 nm. Here, we used the peak intensity at 570 nm to calculate the fluorescence recovery efficiency and quantify the concentration of targets, which exhibited the maximum emission wavelength of Cy3 [35]. When the concentration of the 18S rDNA gene changed between 10^−4^ pM to 10^5^ pM, the fluorescence intensity increased with the increase of the target gene concentration. The fluorescence recovery efficiency showed good linear relationships with the concentration for all six algae, as shown in Figure 4g–l, which offered a quantitative detection basis for the HABs samples containing any of the six algae species using the proposed detection platform. When the ssDNA concentration exceeded 10^−4^ pM (0.1 fM), the 18S rDNA gene’s fluorescence intensity of matched algae was obviously different from that of the other algae. According to the definition of detection limit [36,37] that LOD = 3 × δ/S where δ is the standard deviation of the blank and S is the gradient of the linear regression equation, the detection limit of six algae was calculated and listed in Table S5.

### 3.3. Simulated Samples Detection

**Figure 5:**
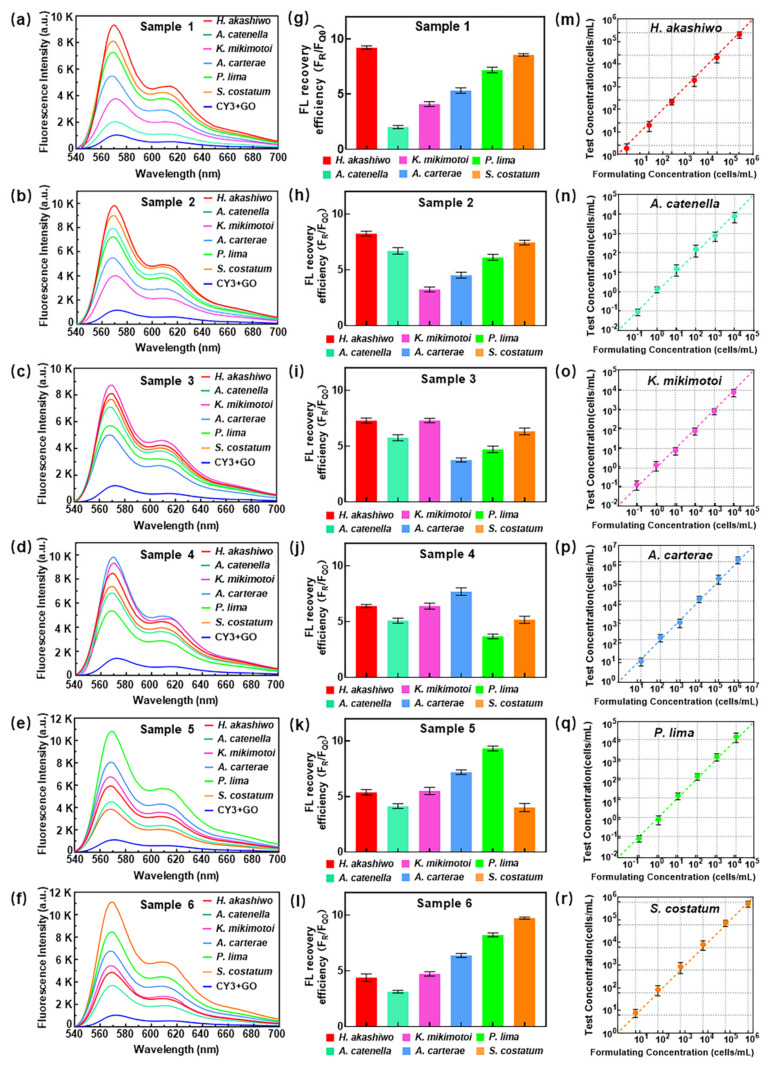
(**a**–**f**) PL spectrum, (**g**–**l**) fluorescence recovery efficiency and (**m**–**r**) the deviation between formulating concentration and test concentration of each HABs.

To evaluate the detection performance of the proposed detection platform in actual seawater, a back-standard experiment was carried out referring to the above linear relationship (Figure 4g–l). The mixed samples were prepared according to the method mentioned in 2.9, and the concentrations of 18S rDNA extracted from six kinds of algal cells were converted to algal cell concentrations shown in Table S6. Figure 5a–l correspond to the PL spectra and fluorescence recovery efficiency of six mixed samples, respectively. It suggested that each component in a certain mixed sample had an obvious fluorescence luminescence peak at 570 nm. The fluorescence intensity, as well as the fluorescence recovery efficiency, were clearly distinguished. Figure 5m–r show the comparison between the formulating concentration and the test concentration in the six mixed samples, demonstrating that the detection platform had a good accuracy response to the detection of HABs 18S rDNA in mixed samples, and it matched the actual concentration in the mixed sample very well.

The main performances of a few representative detection methods are listed in Table 1 together with the proposed method, including primer regime, algae kinds, detection limit and detection time. Compared with other quantitative microalgae detection methods, the proposed detection platform takes much less detection time, most of which is the reaction time of DNA double-stranded hybridization, and the real detection time for each sample is only 0.5–1 s. Additionally, our system achieves lower detection limits with economic and portable equipment, which will benefit detection in the survey location.

## 4. Conclusions

In this work, a microfluidic biochip and corresponding PL detection system were developed to realize ultrasensitive, rapid and quantitative detection of multiple harmful algae. The proposed platform presents excellent selectivity, a linear quantification curve, a large detection range, an ultralow detection limit and a rapid and simple detection process. The entire detection process only consumes 45 min, and it does not require DNA amplification during real sample detection. The widest linear range of fluorescence recovery efficiency was 0.1 fM–100 nM, and the narrowest was 10 fM–100 nM. It is a topic worth exploring in the future that auto-operation could be realized by integrating biochip and PL systems with DNA extraction by taking advantage of automated control operation technology so that researchers can add the samples only and realize a more convenient detection process of HABs. The high detection performance and portable detection system, together with economical cost, enable it to be a promising monitoring tool of algae in HABs early warning and environmental care. Additionally, the biochip-based DNA detection system may be used for nucleic acid detection and analysis in the field of life/health since nucleic acid detection shares similar procedures and requirements.

## Figures and Tables

**Table 1:** Main performance of representative detection methods and the proposed method.

**Method**	**Primers Region**	**Microalgae**	**Detection Limit**	**Time**	**References**
PCR	5.8-ITS rDNA	*Karlodinium armiger*	277 aM target DNA	2.5 h	[38]
mPCR *	ITS rDNA	*Karenia mikimotoi* *Scrippsiella trochoidea*	600 ng/mL target DNA 60 ng/mL target DNA	1–2 h 1–2 h	[39]
RPA *	5.8-ITS rDNA	*2 Ostreopsis*	9 ng/mL target DNA	1–2 h	[40]
E-RCA-LFD *	LSU (D1/D2)	*Karlodinium veneficum*	8 × 10^−6 ^ng/mL DNA	1–2 h	[41]
H-RCA-LFD *	ITS rDNA	*Karenia mikimotoi*	1 × 10^−6^ ng/mL DNA	45 min	[42]
RPA-LFD	ITS rDNA	*Karlodinium veneficum*	1 × 10^4^ ng/mL target DNA	1–2 h	[43]
Microfluidic biochip	18S rDNA	6 harmful algae	10^8^ aM target DNA (1.33 × 10^−6^ ng/mL DNA)	45 min	this work

* mPCR is an abbreviation of multiplex polymerase chain reaction. * RPA is recombinase polymerase amplification. * E-RCA-LFD and H-RCA-LFD represent exponential rolling circle amplification coupled with lateral flow dipstick and hyperbranched rolling circle amplification coupled with lateral flow dipstick, respectively.

## Data Availability

Not applicable.

## Supplementary Materials

The following supporting information can be downloaded at: https://www.mdpi.com/article/10.3390/mi13101759/s1. Figure S1. Layout of double-layer microfluidic biochips. Figure S2. Fabrication process of double-layer microfluidic biochips. Figure S3. Masks of double-layer microfluidic biochips. Figure S4. The double-layer microfluidic biochips. Figure S5. Characterization of graphene oxide. Table S1. Distribution of the six HABs in this paper in Chinese waters. Table S2. Frequency of red tide organisms in China’s offshore waters. Table S3. The probe sequence used in the experiment. Table S4. The concentration of harmful algae in mixed samples. Table S5. Biosensor detection linear equation of six HABs 18S rDNA gene. Table S6. Cells concentration corresponding to 18S rDNA gene concentration. References [44,45] are cited in the supplementary materials.
