# Supplementary material for: A Sensitive and Portable Double-Layer Microfluidic Biochip for Harmful Algae Detection
Source: Micromachines (Basel). 2022 Oct 18;13(10):1759. doi: 10.3390/mi13101759 (PMC9611269; doi:10.3390/mi13101759)
Supplement: Supplementary file 1 [file micromachines-13-01759-s001.zip › micromachines-1980578-supplementary.pdf]

Supporting information

A sensitive and portable double-layer microfluidic biochip for HABs detection

Ping Li[1], Le Qiang[1,2,3], Yingkuan Han[1], Yujin Chu[1], Jiaoyan Qiu[1], Fangteng Song[1], Min Wang[1], Qihang He[1], Yunhong Zhang[1], Mingyuan Sun[1], Caiwen Li[4], Shuqun Sun[4], Yun Liu[4], Lin Han [1,] and Yu Zhang[1,*]*

1 Institute of Marine Science and Technology, Shandong University, Qingdao (266000), China

2 Key Laboratory of Shaanxi Province for Craniofacial Precision Medicine Research, College of Stomatology, Xi'an Jiaotong University

3 Department of Endodontics, Hospital of Stomatology, Xi'an Jiaotong University, Xi'an, China

4 CAS Key Laboratory of Marine Ecology and Environmental Sciences, Institute of Oceanology, Chinese Academy of Sciences, Qingdao 266071, China

* Correspondence: yuzhang@sdu.edu.cn (Yu Zhang), hanlin@sdu.edu.cn (Lin Han)

Ping Li and Le Qiang contributed equally

Content:

Table S1. Distribution of the six HABs in this paper in Chinese waters 3

Table S2. Frequency of red tide organisms in China’s offshore waters 3

Figure S1. Layout of double-layer microfluidic biochips.. 4

Figure S2. Fabrication process of double-layer microfluidic biochips............................. 5

Figure S3. Masks of double-layer microfluidic biochips.. 6

Figure S4. The double-layer microfluidic biochips 6

Figure S5. Characterization of graphene oxide.. 7

Table S3. The probe sequence used in the experiment 8

Table S4. The concentration of harmful algae in mixed samples 9

Table S5. Biosensor detection linear equation of six HABs 18S rDNA gene 10

Table S6. Cells concentration corresponding to 18S rDNA gene concentration 11

Table S1. Distribution of the six HABs in this paper in Chinese waters [1]

Alage	Toxicity	Alarm Value 10^4 cells L^{-1}	Distribution in China		
			Yellow Sea	East Sea	South Sea
Heterosigma akashiwo	*	500	+		
Alexandrium catenella	PSP	50	+	+	+
Amphidinium carterae Hulburt	*	100			+
Karenia mikimotoi	*	100		+	+
Prorocentrum lima	DSP	50		+	+
Skeletonema costatum		1000	+	+	+

Note: *: Toxin production

+: Distributed throughout the region

PSP: Paralytic shellfish poisoning

DSP: Diarrhetic shellfish poison

Table S2. Frequency of red tide organisms in China's offshore waters [2]

Alage	Year Statistics									
	2008	2009	2010	2011	2012	2013	2014	2015	2016	2017
H.akashiwo	2	4	2	2		1	3	2	4	1
A.catenella									2	
K.mikimotoi		1								
A.carterae	3	7	4		19	2	4	3	1	12
P.lima			1							
S.costatum	10	8	6	7	9	6	2	8	8	4

Design of double-layer microfluidic biochip.

The design of the double-layer microfluidic biochip structure is shown in **Figure S1**. The red part represented the upper layer chip for sample adding, the green part expressed the bottom sensor chip structure, and the yellow part was the two-layer shared structure. Double-layer microfluidic biochip contained 12 detection units, each of which was composed of the sample adding part in the upper chip and the sensing part in the bottom. Through the three divided microfluidic channels of the upper chip, GO nanomaterial solution, specific ssDNA probe with fluorescent group, and sample to be tested could be added separately, and then the mixed liquid could be used for nucleic acid hybridization reaction and subsequent detection in the sensing chamber of the bottom chip. In addition, there were micro-channels in the bottom chip to collect the waste liquid of each unit. To achieve the alignment of the upper and lower layers during chip bonding, the micro-channels in the upper chip were designed to penetrate deeply into the space of sensing chamber in the bottom chip.

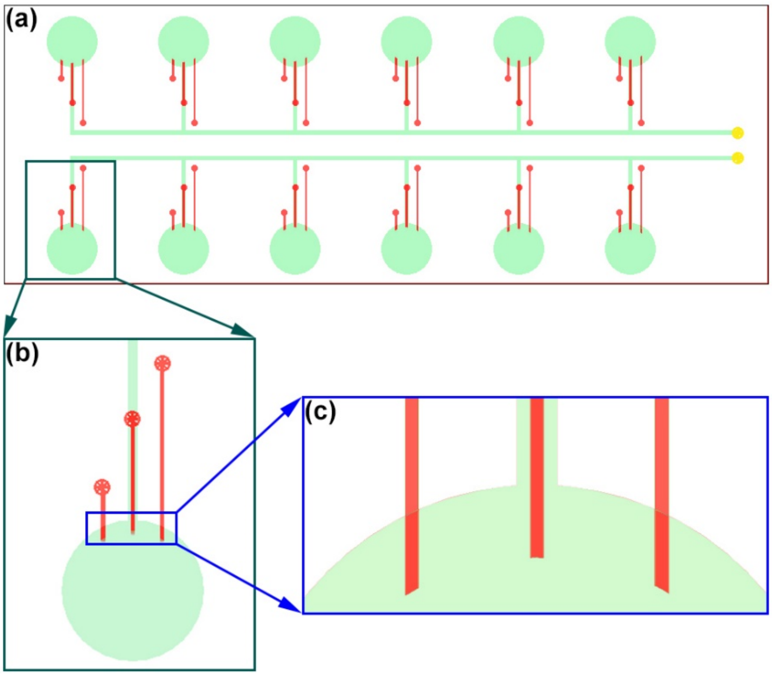

Figure S1. Layout of double-layer microfluidic biochips

(a) General layout of biochip; (b) Layout of every unit; (c) Alignment of double-layer

Manufacturing process of double-layer microfluidic biochip

Figure S2 demonstrates the complete chip fabrication process.

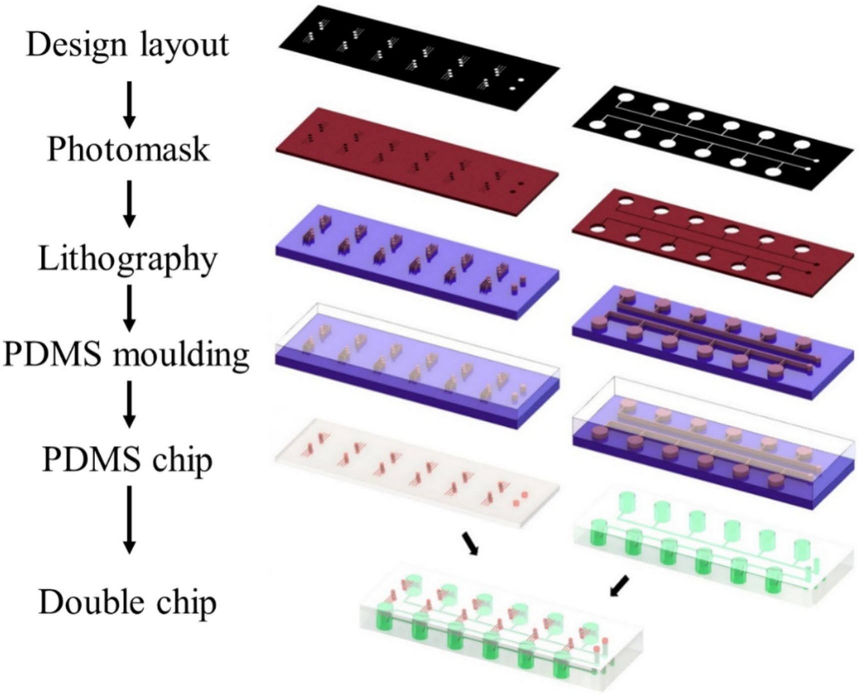

Figure S2. Fabrication process and production of double-layer microfluidic biochips

First of all, the design drawing was made into a film mask strictly in accordance with the design parameters (**Figure S3**). Ultraviolet rays could pass through the transparent part of the mask during photolithography, so that the photoresist under the mask was exposed. This double-layer microfluidic biological chip, with the size of glass slide (7.5 cm × 2.5 cm), integrated sample adding, reaction and detection. It was compact and light, which was convenient to carry when going to sea.

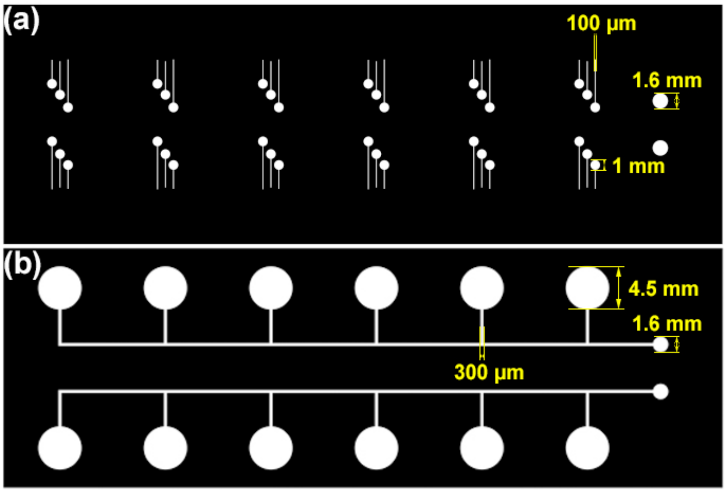

Figure S3. Masks of double-layer microfluidic biochips (a) Upper layer; (b) Bottom layer

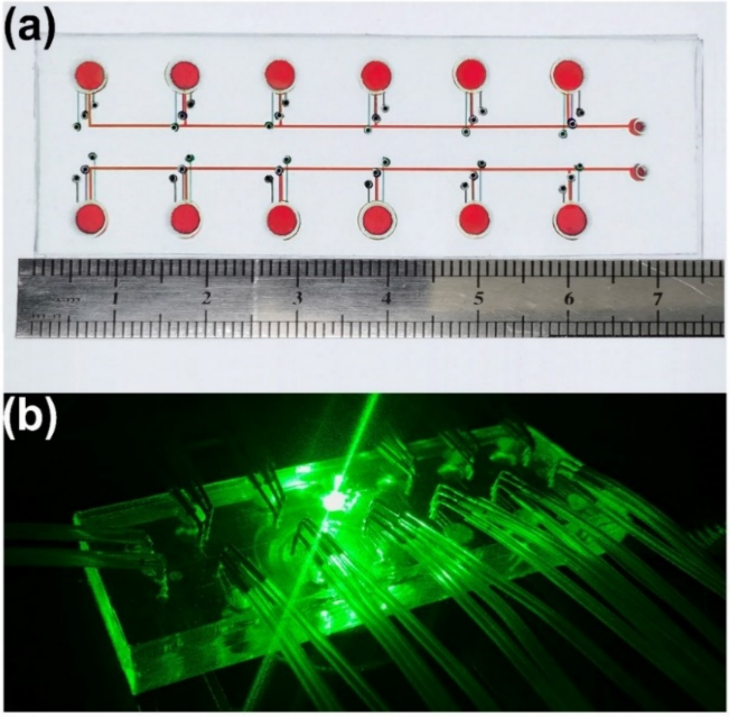

Figure S4. The double-layer microfluidic biochips

(a) Top view (colors are marked with ink); (b) Test view

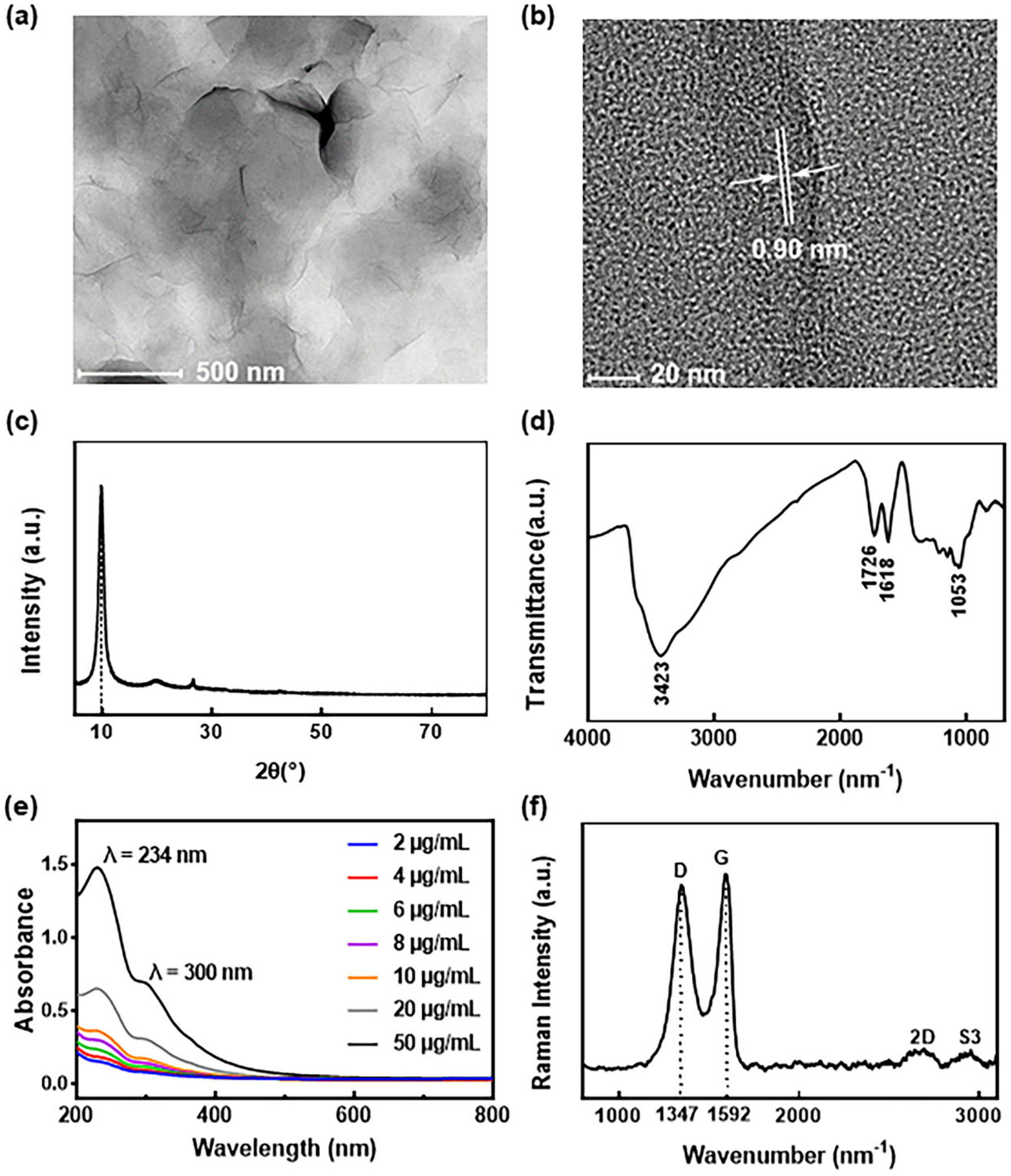

Figure S5. Characterization of graphene oxide (a) TEM image of 500 nm; (b) TEM image of 20 nm; (c) XRD pattern of GO; (d) FTIR spectra of GO; (e) UV-visible absorption spectra of GO at different concentrations; (f) Raman spectra of GO

The probe sequences and part of 18S rDNA sequences of algae used in the experiment are listed in **Table S3**.

Table S3. The probe sequence used in the experiment

	Name	Sequence（5′→3′）	bp
**Probe DNA**	*H.akashiwo*	AACTGCTTCGGCGGACGGGATGTATTTATTAGATGGAAAC-Cy3	40
	A.catenella	CTTCCGTCAATTCCTTTAAGTTTCAGCCTTGCGACCATAC-Cy3	40
	K.mikimotoi	TGGCGAATGAACAGGGATAAGCTCAGCATGGAAATTGGGG-Cy3	40
	A.carterae	GAAGGTATCAGTGTAGCGCGTGCAGCCCAGAACATCTA-Cy3	40
	P.lima	GTCGCAAGGCTGAAACTTAAAGGAATTGACGGAAGGGCAC-Cy3	40
	S.costatum	CGTCAATTTCTTTAAGTTTCAGCCTTGCGACCATACTCCC-Cy3	40
**Target DNA**	*H.akashiwo*	GTTTCCATCTAATAAATACATCCCGTCCGCCGAAGCAGTT	40
	A.catenella	GTATGGTCGCAAGGCTGAAACTTAAAGGAATTGACGGAAG	40
	K.mikimotoi	CCCCAATTTCCATGCTGAGCTTATCCCTGTTCATTCGCCA	40
	A.carterae	TAGATGTTCTGGGCTGCACGCGCGCTACACTGATACCTTC	40
	P.lima	GTGCCCTTCCGTCAATTCCTTTAAGTTTCAGCCTTGCGAC	40
	S.costatum	GGGAGTATGGTCGCAAGGCTGAAACTTAAAGAAATTGACG	40

Table S4. The concentration of harmful algae in mixed samples

Number	Algal cell concentration (cells/mL)					
	H.akashiwo	***A.catenella***	***K.mikimotoi***	***A.carterae***	***P.lima***	***S.costatum***
**1**	2.5×10^{5}	1.01×10^{-1}	9×10^{-1}	1.5×10^{3}	1.22×10^{2}	6.05×10^{4}
**2**	2.5×10^{4}	1.01×10^{4}	9×10^{-2}	1.5×10^{2}	1.22×10^{1}	6.05×10^{3}
**3**	2.5×10^{3}	1.01×10^{3}	9×10^{3}	1.5×10^{1}	1.22×10^{0}	6.05×10^{2}
**4**	2.5×10^{2}	1.01×10^{2}	9×10^{2}	1.5×10^{6}	1.22×10^{-1}	6.05×10^{1}
**5**	2.5×10^{1}	1.01×10^{1}	9×10^{1}	1.5×10^{5}	1.22×10^{4}	6.05×10^{0}
**6**	2.5×10^{0}	1.01×10^{0}	9×10^{0}	1.5×10^{4}	1.22×10^{3}	6.05×10^{5}

Table S5. Detection range, standard curves, and LOD of six HABs 18S rDNA gene

Name	Linear fitting equation	Linear range (pM)	Linear fitting equation of LOD	LOD (aM)	Amplified 18S mass (g/mol)	LOD (ng/mL)
H.akashiwo	Y = 0.9462*(LgX)+5.952	10^{-4}-10^{5}	Y=187.2559X+2.690	108	12316	1.33×10^{-6}
A.catenella	Y = 0.9524*(LgX)+4.319	10^{-3}-10^{5}	Y=99.3892X+1.972	203	12623	2.56×10^{-6}
K.mikimotoi	Y = 0.8587*(LgX)+4.901	10^{-4}-10^{5}	Y=61.3841X+1.870	328	12210	4.00×10^{-6}
A.carterae	Y = 0.8049*(LgX)+5.068	10^{-4}-10^{5}	Y=96.7685X+2.098	208	12380	2.58×10^{-6}
P.lima	Y = 1.082*(LgX)+5.798	10^{-4}-10^{5}	Y=165.6022X+3.188	122	12281	1.50×10^{-6}
S.costatum	Y = 1.173*(LgX)+5.663	10^{-3}-10^{5}	Y=109.0690X+1.823	185	12623	2.34×10^{-6}

LOD= 3 × δ/S where δ is the standard deviation of the blank and S is the gradient of the linear regression equation, the average background = 1063.41733(a.u), δ = 0.00672.

Molecular weight of DNA=(A_n×313.2) + (T_n×304.2) + (C_n×289.2) + (G_n×329.2) + 79.0, where A_n, T_n, C_n and G_n represent the number of A, T, C and G nucleobase respectively, meanwhile, the increase of 79 is due to consideration of the mass of 5 '-end monophosphate.

Take *H.a kashiwo* as an example:

LOD=3 × δ/S =3×0.00672/187.2559=108 (aM)

The amplified 18S rDNA strand length is 40, the numbers of A, T, C, and G nucleobase are 11, 11, 12 and 6 respectively.

Amplified 18S mass= (11×313.2) + (11×304.2) + (12×289.2) + (6×329.2) + 79.0=12316 (g/mol)

LOD=108 (aM) ×12316 (g/mol) = 1.33×10^{-6} (ng/mL)

Table S6. Cells concentration of six HABs corresponding to 18S rDNA gene concentration

18S rDNA	Algal cell concentration (cells/mL)	18S rDNA concentration (μg/mL)	18S rDNA length (bp)	18S rDNA molecular mass (g/mol)	18S rDNA of per cell (pmol/cells)	cell concentration of 1 pM 18S rDNA (cells/mL)
H.akashiwo	2.50×10^{5}	3.69	1695	1029701	1.432×10^{-5}	69.798
A.catenella	1.01×10^{4}	0.61	2111	1282379.4	4.679×10^{-5}	21.373
K.mikimotoi	9.00×10^{3}	0.72	1423	864488.2	9.254×10^{-5}	10.806
A.carterae	1.50×10^{6}	4.04	2080	1263550	2.134×10^{-6}	468.676
P.lima	1.22×10^{4}	1.65	1910	1160292	1.167×10^{-4}	8.566
S.costatum	6.05×10^{5}	2.83	1781	1081937.4	4.316×10^{-6}	231.644

The Qubit™ 3.0 fluorimeter was used to measure the concentrations of 18S rDNA extracted from six kinds of algae cells with known concentrations, and the DNA concentrations were converted to the algae cell concentrations.

Reference

[1] HY/T 069-2005; State Oceanic Administration (SOA). Technical Specification for Red Tide Monitoring in China. China National Standardization Management Committee: Beijing, China, 2005.

[2] Zhang SF, Wang Q, Guan CY, Shen XX, Li RL. Study on the Occurrence Law of Red Tide and lts Influencing Factors in the Offshore Waters of China from 2001 to 2017 [J]. Journal of Peking University (Natural Science Edition),2020,56(06): 1129-1140.
